# Purification, Gene Cloning, and Biochemical Characterization of a β-Glucosidase Capable of Hydrolyzing Sesaminol Triglucoside from *Paenibacillus* sp. KB0549

**DOI:** 10.1371/journal.pone.0060538

**Published:** 2013-04-10

**Authors:** Arun Nair, Akika Kuwahara, Akihiro Nagase, Haruhiko Yamaguchi, Tatsuya Yamazaki, Miho Hosoya, Ayano Omura, Kunio Kiyomoto, Masa-atsu Yamaguchi, Takefumi Shimoyama, Seiji Takahashi, Toru Nakayama

**Affiliations:** 1 Kiyomoto Co. Ltd, Nobeoka, Miyazaki, Japan; 2 Laboratory of Applied Life Chemistry, Department of Biomolecular Engineering, Graduate School of Engineering, Tohoku University, Sendai, Miyagi, Japan; 3 Faculty of Health and Nutrition, Department of Food Science for Health, Minami Kyushu University, Miyakonojo, Miyazaki, Japan; Centro Nacional de Biotecnologia - CSIC, Spain

## Abstract

The triglucoside of sesaminol, *i.e.*, 2,6-*O*-di(β-D-glucopyranosyl)-β-D- glucopyranosylsesaminol (STG), occurs abundantly in sesame seeds and sesame oil cake and serves as an inexpensive source for the industrial production of sesaminol, an anti-oxidant that displays a number of bioactivities beneficial to human health. However, STG has been shown to be highly resistant to the action of β-glucosidases, in part due to its branched-chain glycon structure, and these circumstances hampered the efficient utilization of STG. We found that a strain (KB0549) of the genus *Paenibacillus* produced a novel enzyme capable of efficiently hydrolyzing STG. This enzyme, termed PSTG, was a tetrameric protein consisting of identical subunits with an approximate molecular mass of 80 kDa. The PSTG gene was cloned on the basis of the partial amino acid sequences of the purified enzyme. Sequence comparison showed that the enzyme belonged to the glycoside hydrolase family 3, with significant similarities to the *Paenibacillus* glucocerebrosidase (63% identity) and to Bgl3B of *Thermotoga neapolitana* (37% identity). The recombinant enzyme (rPSTG) was highly specific for β-glucosidic linkage, and *k*_cat_ and *k*_cat_/*K*_m_ values for the rPSTG-catalyzed hydrolysis of *p*-nitrophenyl-β-glucopyraniside at 37°C and pH 6.5 were 44 s^−1^ and 426 s^−1^ mM^−1^, respectively. The specificity analyses also revealed that the enzyme acted more efficiently on sophorose than on cellobiose and gentiobiose. Thus, rPSTG is the first example of a β-glucosidase with higher reactivity for β-1,2-glucosidic linkage than for β-1,4- and β-1,6-glucosidic linkages, as far as could be ascertained. This unique specificity is, at least in part, responsible for the enzyme’s ability to efficiently decompose STG.

## Introduction

**Figure 1:**
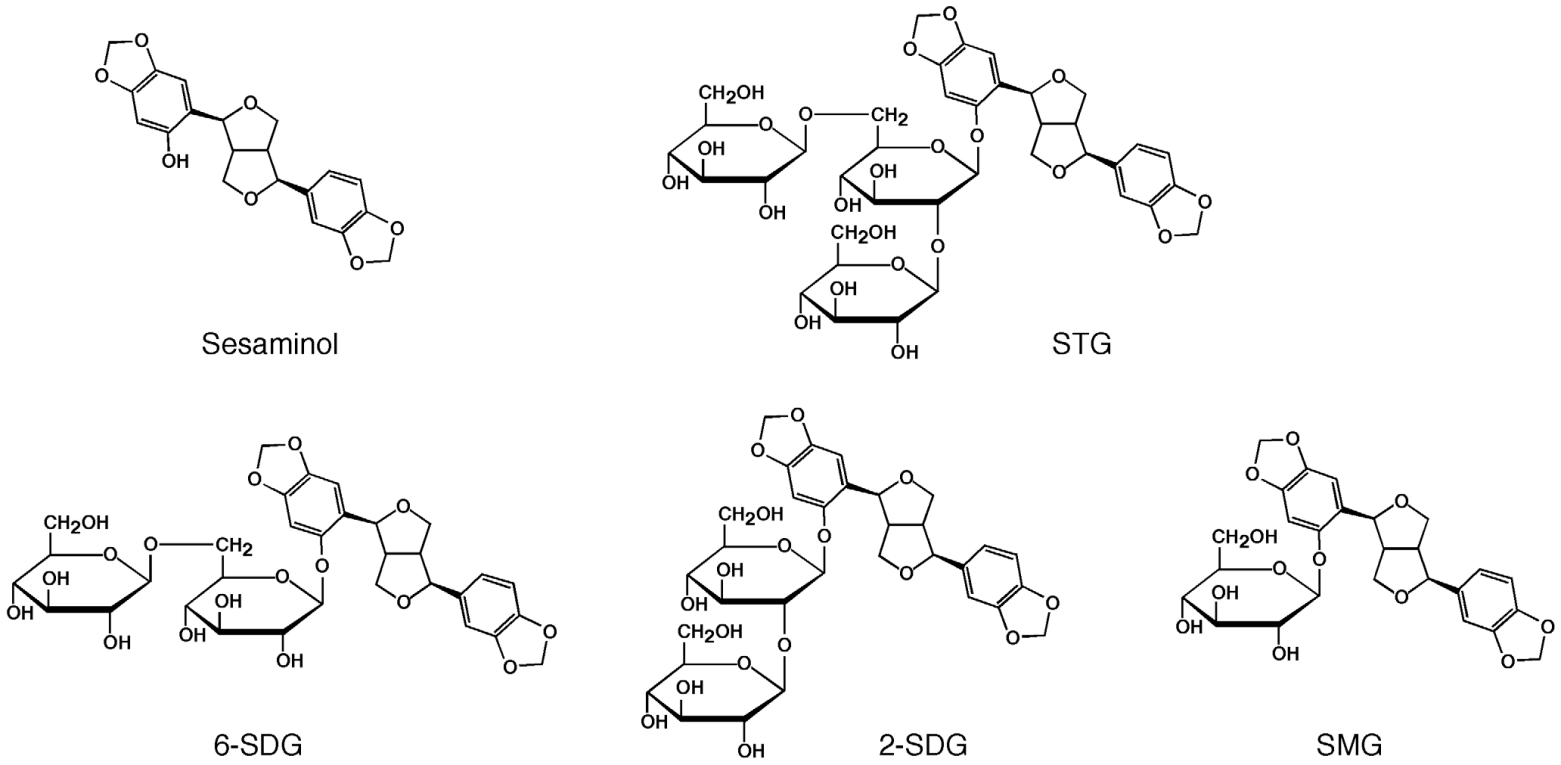
Structures of sesaminol-related glucosides. STG, 2,6-*O*-di(β-D-glucopyranosyl)-β-D-glucopyranosylsesaminol; 2-SDG, 2-*O*-(β-D -glucopyranosyl)-β-D-glucopyranosylsesaminol; 6-SDG, 6-*O*-(β-D -glucopyranosyl)-β-D-glucopyranosylsesaminol, and SMG, β-D -glucopyranosylsesaminol.

Sesaminol (Fig. 1) is one of the lignans identified in sesame oils, and it displays potent antioxidant activity [1]–[3]. Sesame oils produced from both unroasted and roasted sesame seeds are more stable than other vegetable oils, and this arises from the presence of sesaminol aglycons (i.e., sesaminol and its isomers). Sesaminol also shows a variety of bioactivities that are beneficial to human health, serving as a potent inhibitor of the oxidation of low-density lipoproteins [3], [4] and showing anti-tumor activity by the induction of apoptosis in human lymphoid leukemia cells [5].

Sesame seeds contain large amounts of lignans in the form of both aglycons and their glycosides. The most abundant lignans in sesame seeds include sesaminol triglucosides [2,6-*O*-di(β-D-glucopyranosyl)-β-D-glucopyranosylsesaminol, termed STG; Fig. 1], sesamin, sesamolin, and the glucosides of pinoresinol [6], [7]. It is important to note that STG displays only a negligible level of antioxidant activity *in vitro* [8], [9]. Moreover, the formation of bioactive sesaminol in sesame oils does not arise from the hydrolysis of STG; it is produced from sesamolin through acid catalysis during the bleaching step of the refining process of sesame oil production [10], [11]. STG occurs abundantly in sesame oil cake, and is produced as a by-product of sesame oil production in a large amount and can serve as an inexpensive source for the industrial production of sesaminol. Although the production of sesaminol through the hydrolysis of STG extracted from sesame oil cake appears to be an ideal route to meet the growing demand for this natural antioxidant, STG is highly resistant to the hydrolytic action of β-glucosidases, probably due to its branched-chain glycon structure and sterically hindered aglycon structure [6], [7]. In fact, only a few examples of the inefficient production of sesaminol and related products from STG have been reported. Antioxidative lignans, including sesaminol 6-catechol, were produced from sesaminol triglucoside by culturing with the genus *Aspergillus* [8]. Sesaminol was also isolated from de-fatted sesame seeds by treatment with a fungus, *Absidia corymbifera* [5].

The present study searched for a microorganism that produces an enzyme capable of hydrolyzing STG to produce sesaminol. A bacterial strain, KB0549, isolated from sesame oil cake, was found to produce a novel β-glucosidase, which was able to efficiently hydrolyze all of the glucosidic linkages in the STG molecule. Phylogenetic analysis showed that the strain KB0549 belonged to the genus *Paenibacullus*. We describe here the purification, molecular cloning, heterologous expression, and characterization of this enzyme, STG-hydrolyzing β-glucosidase from *Paenibacullus* sp. (termed PSTG).

## Materials and Methods

### Chemicals

*p*-Nitrophenyl-**(**pNP-)β-D-glucopyranoside (pNP-β-Glc), pNP-α-D-gluco- pyranoside, pNP-β-D-cellobioside pNP-β-D-xylopyranoside, and pNP-β-D-galacto- pyranoside were obtained from Nacalai Tesque, Kyoto, Japan. pNP-β-D-Fucopyranoside was obtained from Santa Cruz Biotechnology (Santa Cruz, CA) and pNP-*N*-acetyl-β-D-glucosaminide was from Sigma-Aldrich Japan (Tokyo, Japan). Sophorose was purchased from Extrasynthese (Genay Cedex, France), and cellobiose and gentiobiose were from Wako Pure Chemical Industries (Tokyo, Japan). Stereo isomers of sesaminol (2-episesaminol, 6-episesaminol, and diasesaminol) were purchased from Nagara Science (Gifu, Japan).

STG was extracted with water from sesame oil cake produced from a local sesame-oil manufacturer in Kagawa, Japan. The extract was applied to a column (2 cm×40 cm) of HP20 (Mitsubishi Chemicals, Tokyo, Japan) equilibrated with 30% (by volume) ethanol in water. STG was eluted with 50% (by volume) ethanol in water, evaporated to dryness, and dissolved with a minimum volume of water. The crude STG concentrate was then subjected to preparative reversed-phase high performance liquid chromatography (HPLC) using a Gilson 305 HPLC system, as follows: column, Mightysil RP-18GP (20×250 mm, Kanto Chemicals, Tokyo, Japan); flow rate, 4.0 ml/min; solvent and development, isocratic elution with 0.2% (v/v) acetic acid in a 3∶7 (v/v) mixture of acetonitrile and H_2_O; and, detection of absorbance at 290 nm. 6-*O*-(β-D-Glucopyranosyl)-β-D-glucopyranosylsesaminol (6-SDG, Fig. 1), β-D-glucopyranosylsesaminol (SMG, Fig. 1), and sesaminol were prepared by partial hydrolysis of STG by PSTG and isolated by means of reversed-phase HPLC (for the standard HPLC conditions, see Enzyme assay, Method I). The structures of STG and SDGs were confirmed by ^1^H-NMR analyses [6], [7]. STG, SDGs, SMG, and sesaminol were also identified by matrix-assisted laser desorption/ionization time of flight mass spectrometry (MALDI-TOF MS) on an AXIMA-CFR plus spectrometer (Shimadzu, Kyoto, Japan), as follows: STG, *m/z* 856 [M+Na^+^]; SDGs, *m/z* 694 [M+Na^+^]; SMG, *m/z* 532 [M+Na^+^]; and, sesaminol, *m/z* 370 [M·]. For MALDI-TOFMS analysis, 10 mg/ml 2,5-dihydroxybenzoic acid (for STG, SDGs, and SMG) [or 10 mg/ml (w/v) α-cyano-4-hydroxycinnamic acid (for sesaminol)], 1% (by volume) trifluoroacetic acid, and 9% (v/v) acetonitrile in H_2_O was used as a matrix solution. For all other chemicals, the purest reagents available were used.

### Bacterial Strains

*Paenibacillus* sp. strain KB0549, which was a stock culture from the Kiyomoto Co., Miyazaki, Japan, was deposited to the International Patent Organism Depositary, National Institute of Advanced Industrial Science and Technology, Tsukuba, Ibaraki, Japan, under accession number FERM AP-21057. Cells of the strain KB0549 were grown with shaking at 37°C for 3 days in a broth (pH 6.5) containing 0.5% (w/v) Bacto Peptone (BD, New Jersey), 0.25% yeast extract, and 0.025% STG (medium 1). *Escherichia coli* strains DH5α (Takara Bio; Shiga, Japan) and BL21-AI (Life Technologies Japan; Tokyo, Japan) were used for the cloning and expression of the *PSTG* gene, respectively.

### 16S rRNA Sequence Analysis

*Paenibacillus* sp. KB0549 cells were grown in a medium (pH 7.0) containing 1% soluble starch, 0.5% peptone, and 0.5% yeast extract (medium 2). Isolation of the genomic DNA from *Paenibacillus* sp. KB0549 cells was performed essentially as described by Murray and Thompson [12]. The 16S rDNAs in the genome were amplified by PCR using ExTaq DNA polymerase (Takara Bio) and universal (U) or bacteria-specific (B) 16S rDNA primers: B27f primer (5′-AGAGTTTGATCCTGGCTCAG-3′) [13]; U515f primer (5′-GTGCCAGCMGCCGCGG-3′) [14]; U533r primer (5′-TTACCGCGGCKGCTGRCAC-3′) [14]; B968f primer (5′-AACGCGAAGAACCTTAC-3′) [14]; U1492r primer (5′-GGTTACCTTGTTACGACTT-3′) [14]. The amplified 1445-bp DNA fragments were separated in a TAE (20 mM Tris–acetate, pH 7.4, 10 mM acetate, and 0.5 mM EDTA) agarose gel. After electrophoresis, the gel was stained with ethidium bromide and visualized with a UV illuminator. The amplified 16S rDNA band was purified by using a QIAquick PCR purification kit (QIAGEN, Hilden, Germany). The fragments were then sequenced with a CEQ2000 DNA sequencer (Beckman-Coulter, Fullerton, CA). The obtained sequences were classified by comparison with those in public DNA databases.

### Enzyme Assays

#### Method I

The enzymatic hydrolysis of STG was monitored by HPLC. The standard reaction mixture (final volume, 500 µl) contained 1.15 mM STG, 75 mM potassium phosphate buffer, pH 7.0, and the enzyme (typically, 0.15 µM). The mixture without the enzyme was brought to 37°C. The reaction was started by the addition of the enzyme. At an appropriate time interval, an aliquot of the mixture was withdrawn and the reaction was stopped by heating the mixture at 100°C for 3 min. Analysis of STG, SDGs, SMG, and sesaminol in the reaction mixture (20 µl) was performed using a Shimadzu LCsolution HPLC system, as follows: column, Mightysil GP RP-18GP (ODS) (4.6×150 mm); flow rate, 0.7 ml/min; solvent A, 0.1% (v/v) trifluoroacetic acid in a 2∶8 (v/v) mixture of acetonitrile and H_2_O; and, solvent B, 0.1% trifluoroacetic acid in a 8∶2 (v/v) mixture of acetonitrile and H_2_O. After injection of the mixture onto the column, which was equilibrated with 10% B (v/v), the column was initially developed isocratically with 10% B for 3 min, followed by a linear gradient from 10% B to 100% B in 24 min. The column was then washed isocratically with 100% B for 3 min, followed by a linear gradient from 100% B to 10% B in 1 min. There was a 5 min delay before the next injection to ensure re-equilibration of the column. The chromatograms were obtained with detection at 290 nm. Typical retention times of sesaminol and its glucosides under the standard HPLC conditions were as follows: STG, 8.52 min; 6-SDG, 10.88 min; 2-SDG, 11.08 min; SMG, 13.60 min; and sesaminol, 20.92 min. These compounds were also identified by MALDI-TOF MS analysis, as described above. One katal (kat) of enzyme was defined as the amount of enzyme that catalyzes the consumption of 1 mol of substrate per second. The specific activity was expressed as kat/mg of protein.

#### Method II

For the kinetic analysis of enzymatic hydrolysis of pNP-glycosides, the standard assay mixture contained varying amounts of one of the pNP-glycosides, 5 µmol of potassium phosphate buffer, pH 7.0, and the enzyme in a final volume of 0.5 ml. The mixture without the enzyme was brought to 37°C. The reaction was started by the addition of the enzyme, and changes in absorbance at 405 nm were recorded in 1 cm path-length cells with a Hitachi double-beam spectrophotometer (model U-2000 or model 2910; Hitachi High-Technologies, Tokyo, Japan). The extinction coefficient for *p*-nitrophenol under these conditions was 8,900 cm^−1^ M^−1^ [15]. *K*_m_ and *k*_cat_ values and their standard errors were estimated by fitting the initial velocity data to the Michaelis-Menten equation by nonlinear regression methods [16].

#### Method III

Glucose that formed in the reaction mixture was determined by the method of Miwa et al. [17] with a kit (Wako Glucose CII Test, Wako Pure Chemical Industries). The analysis was performed in accordance with the guidelines provided by the manufacturer. The blank did not contain the enzyme.

### Purification of PSTG

All purification procedures were carried out at 4°C. The cells were harvested by centrifugation at 12,000×*g* for 20 min. The cells (5.3 g, wet wt) were re-suspended in a final volume of 30 ml of 10 mM potassium phosphate buffer, pH 6.5 (termed buffer A) and disrupted by sonication using a Branson Sonifer Cell Disruptor (Apollo Ultrasonics, York, UK) at a constant duty cycle (1 min for 10 cycles), followed by centrifugation at 10,000×*g* for 30 min. The supernatant was dialyzed overnight against buffer A.

#### ANX sepharose

The enzyme solution was applied to a column (1.6 cm×10 cm) of ANX Sepharose 4 Fast Flow (high sub) (GE Healthcare UK, Buckinghamshire, UK) equilibrated with buffer A. β-Glucosidase activity bound to the column was eluted with a linear gradient (0–1.0 M in 8 column volumes) of NaCl in buffer A. Fractions containing the enzyme activity were combined and concentrated, using Amicon Ultra-15 Centrifugal Filter Devices (30,000 MWCO) (Millipore, Billerica, MA, USA), to appropriate volumes and extensively dialyzed against buffer A.

#### Q sepharose

The enzyme solution was applied to a column (1.6 cm×10 cm) of Q Sepharose Fast Flow (1.6 cm×10 cm, GE Healthcare UK) equilibrated with buffer A. The β-glucosidase activity was eluted with a linear gradient (0–1.0 M in 8 column volumes) of NaCl in buffer A. Fractions containing the enzyme activity were combined and concentrated, using an Amicon centrifugal filter device, to appropriate volumes and extensively dialyzed against buffer A.

#### Hydroxyapatite

The enzyme solution was applied to a column (1.0 cm×6.4 cm) of Bio-Scale CHT5-I hydroxyapatite (Bio-Rad Laboratories, Tokyo, Japan), which was previously equilibrated with buffer A. The enzyme activity was eluted with a linear gradient (10 mM–500 mM) of potassium phosphate in 12 column volumes. Active fractions were combined and dialyzed against buffer A.

#### Mono Q

The enzyme solution was applied to a Mono Q column (1.0 cm×10 cm; GE Healthcare UK) equilibrated with buffer A and eluted with buffer B, with a linear gradient (0.1 M–0.5 M) of NaCl in 10 column volumes. Active fractions were combined.

#### Phenyl sepharose

The enzyme solution was applied to a column (1.6 cm×2.5 cm) of Phenyl Sepharose High Performance (GE Healthcare UK) equilibrated with buffer A containing 20% v/v ethyleneglycol. The activity was eluted with a linear gradient (20%–50%, v/v) of ethyleneglycol in buffer A in 8 column volumes. The active fractions were pooled as the final product.

### Protein Chemical Analyses

Sodium dodecyl sulfate-polyacrylamide gel electrophoresis (SDS-PAGE) was carried out using 10% gels with the Laemmli method [18]. Protein was visualized by silver staining or staining with Coomassie brilliant blue R-250. Protein bands in the SDS-PAGE gels were transferred to a polyvinylidene difluoride membrane (Millipore, Billerica, MA) by electroblotting, and the membrane was stained with Coomassie brilliant blue R-250. The stained PSTG band in the membrane was excised with dissecting scissors and subjected to automated Edman degradation using a Hewlett-Packard G1005A Protein Sequencer (Hewlett-Packard, Palo Alto, CA). To determine the internal amino acid sequences of the enzyme, the protein in the SDS-PAGE gels was digested with *Achromobacter* lysylendopeptidase (Wako Pure Chemical Industries) at 35°C for 20 h (pH 8.5), and the resultant peptides were separated by a reversed-phase HPLC system, as described previously [19]. The *N*-terminal amino acid sequences of the purified peptides were determined as described above.

### Cloning of *PSTG* Gene from *Paenibacillus* sp. KB0549

A 1.5-kb fragment was amplified from the genomic DNA (see above) of strain KB0549 by PCR, using degenerate primers 5′-TCACAAATGACRTTAGAAGAAAAGGC-3′ and 5′-ATCGCTTAAYTGNACCGGGAANGTYTC-3′, which were designed on the basis of the amino terminal and internal sequences of the purified enzyme (see RESULTS section). The PCR product, 1.5 kbp in length, was gel-purified and cloned into a pCR 2.1-TOPO vector (Life Technologies Japan). The recombinant plasmids were used to transform *E*. *coli* DH5α competent cells. The transformants were selected on LB plates containing 100 µg/ml kanamcyin. The recombinant plasmids were isolated and sequenced on a Beckman CEQ 2000 DNA Analysis System (Beckman Coulter, Fullerton, CA). Flanking sequences of the 1.5-kbp genomic sequence, including partial *PSTG* coding regions, were amplified by a DNA-Walking Annealing Control Primer PCR strategy [20] using a DNA Walking *SpeedUp* Premix kit (Seegene, Del Mar, CA). After the sequencing of the franking sequences, a possible full-length PSTG gene, 2.3 kbp in length, was amplified from genomic DNA by PCR using primers Si80F (5′-CACCATGAGTGAACGACGGGATTTGAAAGCACTG-3′) and Si80R5 (5′-TCAGCCGTTCAAATATTCAAGCAGCTTGC-3′) or Si80R6 (5′-GGATATGACGTTGTAACATGATCAGCCG-3′). The amplified DNA fragment was gel-purified, cloned into a pENTR/TEV/D-TOPO vector (Life Technologies Japan), and sequenced to confirm its nucleotide sequence.

### Heterologous Expression and Purification of the Recombinant PSTG (rPSTG)

The amplified DNA fragment was then transferred into pDEST17 (Life Technologies Japan), and the resultant recombinant plasmid was used to transform *E. coli* BL21-AI cells. After transformant cells were pre-cultured at 37°C for 16 h in Luria-Bertani broth medium containing 100 µg/ml ampicillin, the culture (50 ml) was inoculated into the same medium (2500 ml). After cultivating the cells at 23°C until the optical turbidity at 600 nm of the culture reached 0.5–0.6, L-(+)-arabinose was added to the medium at a final concentration of 1.0 mM, followed by overnight cultivation at 20°C. All subsequent operations were conducted at 0–4°C. The cells were harvested by centrifugation (15 min, 5,000×*g*) and resuspended in 10 mM potassium phosphate buffer, pH 7.0, containing 1 mg/ml lysozyme, 0.5 mM phenylmethylsulfonylfluoride, and 0.05% 3-[(3-cholamidopropyl)dimethylammonio]-1-propanesulfonate). The cell suspension was chilled on ice for 1 h and then sonicated using a Branson Sonifer Cell Disruptor at a constant duty cycle (10 sec for 5 cycles). The resultant cell debris was removed by centrifugation (15 min, 5,000×*g*) and filtration with a 0.22-µm filter. The enzyme solution was applied to a 1-ml HisTrap HP column (GE Healthcare UK) equilibrated with buffer A containing 30 mM imidazole. The column was washed with the equilibration buffer, and the enzyme was eluted with buffer A containing 200 mM imidazole. The column-bound fractions were concentrated, desalted, and substituted with buffer A using Amicon Ultra-15 Centrifugal Filter Devices. The concentration of the recombinant enzyme, rPSTG, was determined using the absorption coefficient of rPSTG, *ε*_280_, of 75135 M^−1^ cm^−1^, which was calculated from the deduced amino acid sequence.

### Determination of Native Molecular Mass

The native molecular mass of rPSTG was estimated by gel filtration chromatography on a Superdex 200 column (1.0 cm×30 cm), equilibrated with 0.02 M potassium phosphate buffer, pH 7.0, containing 0.15 M NaCl. The column was developed with the equilibration buffer at a flow rate of 0.24 ml/min by monitoring the absorbance at 280 nm. For calibration, ferritin (*M*r 440,000), catalase (*M*r 232,000), conalbumin (*M*r 75,000), and carbonic anhydrase (*M*r 27,000), all of which were obtained from GE Healthcare UK, were chromatographed under the same conditions.

### pH-Activity Profiles

The enzymatic hydrolysis of pNP-β-Glc (final concentration, 1.0 mM) was assayed by Method II with the following modifications. The reaction mixtures contained 20 mM of one of the following buffers: pH 4.0–5.5, sodium acetate; pH 5.5–7.5, potassium phosphate; pH 7.5–8.5, HEPES-NaOH; pH 8.5–10.0 glycine-NaOH; changes in absorbance at 348 nm (an isosbestic point of *p*-nitrophenol) were recorded.

### Temperature-activity Profiles

The enzymatic hydrolysis of pNP-β-Glc (final concentration, 1.0 mM) was assayed at 5–75°C at 5°C intervals essentially by Method II.

### Stability Studies

For the thermal stability studies, the enzyme (rPSTG) was incubated in standard buffer at 5−50°C at 5°C intervals. After incubation for 30 min, aliquots were withdrawn, placed into tubes in ice, and assayed for remaining activity essentially by Method II using pNP-β-Glc (final concentration, 1.0 mM) as a substrate. For the pH stability studies, the enzyme (rPSTG) was incubated at 4°C for 15 h in one of the following buffers (final concentration, 20 mM): pH 4.0–5.5, sodium acetate; pH 5.5–7.5, potassium phosphate; pH 7.5–8.5, HEPES-NaOH; or, pH 8.5–10.0, glycine-NaOH. After incubation, aliquots were withdrawn and assayed for remaining activity, as described above.

## Results

### Isolation and Taxonomic Characterization of the Strain KB0549

Samples collected from decaying sources of sesame oil cake were subjected to extraction with chloroform and the resultant extracts were analyzed for sesaminol by HPLC. One of the samples (No. 0549) was found to contain a trace amount of sesaminol. Thus, a small amount of the No. 0549 sample was suspended in a medium (yeast extract 2.5 g, peptone 5.0 g, glucose 1.0 g, and 100 g sterile sesame oil cake per liter) and cultured for 72 h at 37°C with shaking. After confirming the production of sesaminol in the culture broth, microorganisms that grew on an agar medium 1 (see Materials and methods section) containing 0.025% STG were isolated from this culture, and their ability to degrade STG was further analyzed by HPLC (Method I). The only isolate displaying the STG-degrading capacity, strain KB0549, was a Gram-positive rod. To find the phylogenetic relationship of the bacterium with known bacteria, the genomic DNA of the bacterium was extracted and the DNA coding for 16S rRNA was amplified by PCR. The nucleotide sequence of the amplified DNA was determined (Genbank/EMBL/DDBJ accession number, AB567661) and compared with the available 16S rDNA sequences. The highest sequence similarity was found with 16S rDNA sequences of *Paenibacillus cookii* LMG18419^T^ (97%), *P. cineris* LMG18^T^ (94%), and *P. favisporus* GMP01^T^ (95%), suggesting that strain KB0549 should be classified into the genus *Paenibacillus*. However, judging from low similarity (<98%) to known species of *Paenibacillus* on the basis of a 16S rDNA sequence, it may be a novel species. Detailed taxonomic studies of strain KB0549 will be reported elsewhere.

### Purification and Partial Amino Sequences of PSTG

**Figure 2:**
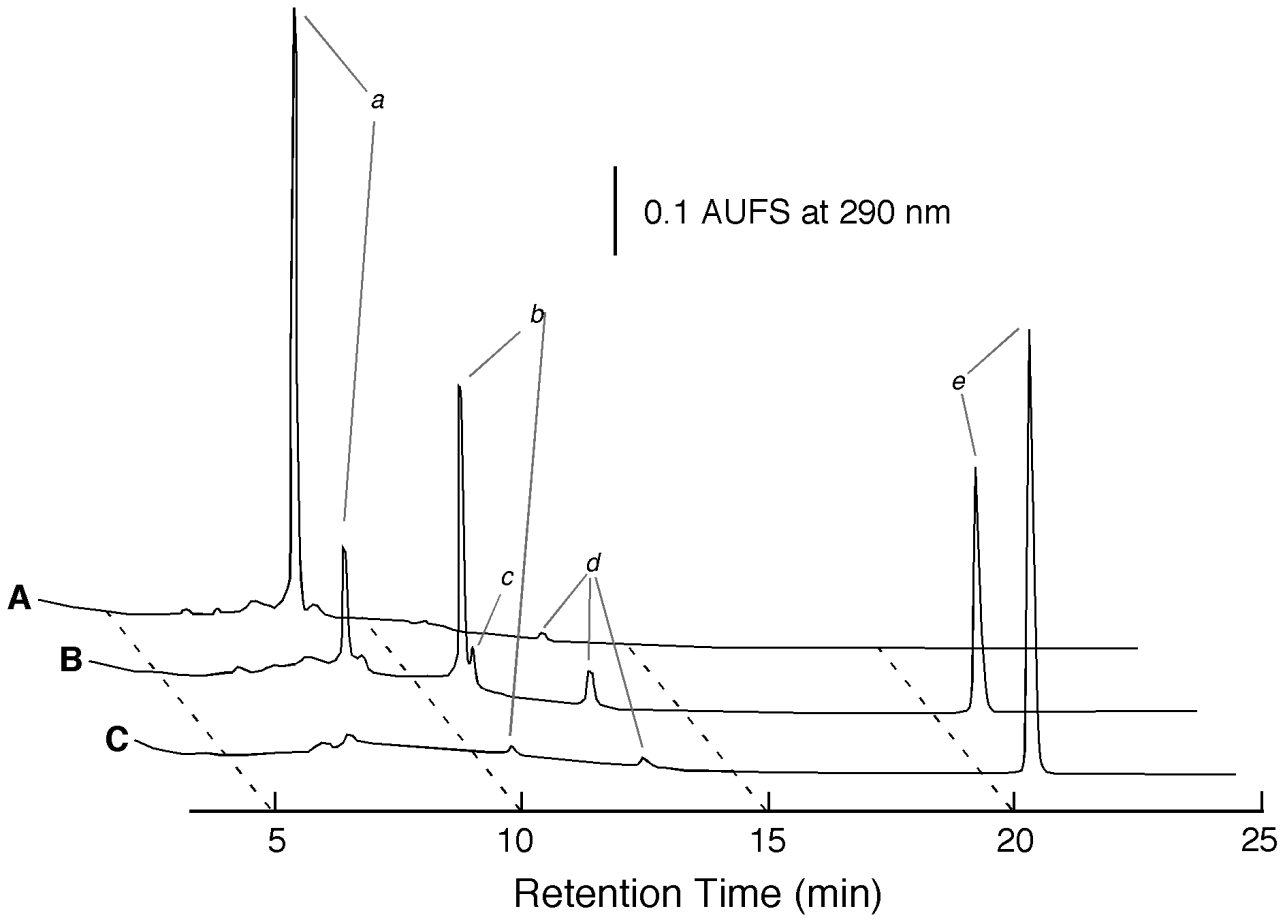
Reversed-phase HPLC analysis of STG hydrolysis with the crude extract of the KB0549 cells. The reaction was carried out using method I (see Enzyme assays; final protein concentration, 0.22 mg/ml). Chromatogram A represents that of zero time of the reaction and chromatograms B and C are those for 1 and 3 h after the initiation of the reaction, respectively. Peak *a*, STG; peak *b*, 6-SDG; peak *c*, 2-SDG; peak *d*, SMG; and peak *e*, sesaminol.

**Figure 3:**
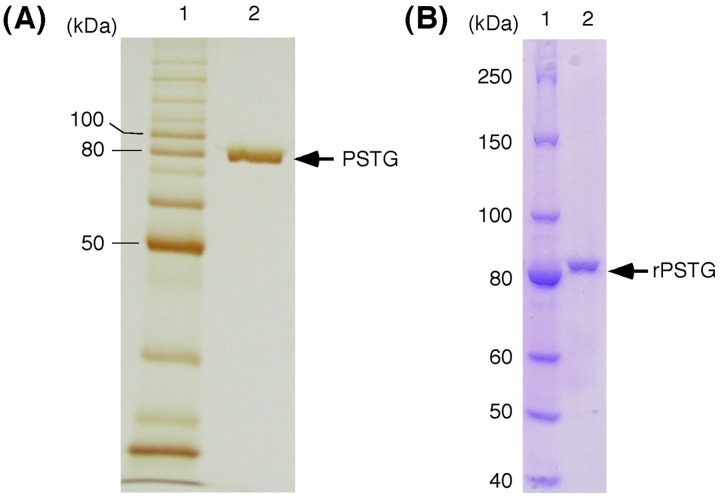
SDS-PAGE analysis of purified enzymes [(A), PSTG; (B), rPSTG]. Lane 1, molecular mass markers; lane 2, purified enzyme.

**Figure 4:**
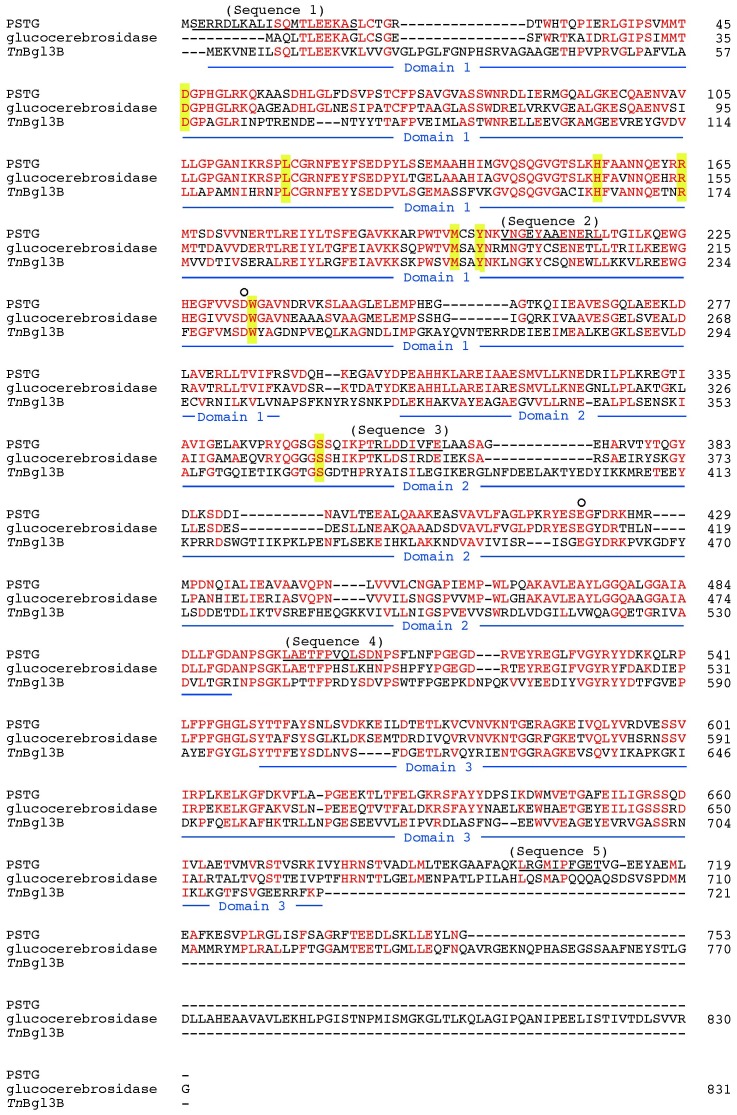
Alignment of the deduced amino acid sequence of PSTG protein with those of enzymes (TS12 glucocerebrosidase [**24**] and *Tn*Bgl3B β-glucosidase [**28**]) belonging to the GH3 family. Amino acid residues identical to those of PSTG are shown in red. Peptides identified from purified PSTG are underlined (sequences 1–5). The putative catalytic residues of PSTG, Asp233 and Glu421, corresponding to those identified in TS12 glucocerebrosidase by affinity labeling studies [25] and in *Tn*Bgl3B by X-ray crystallography [28], are shown with open circles above the PSTG sequence. Putative sugar-binding amino acid residues at subsite –1 of PSTG and glucocerebrosidase, predicted from the crystal structure of *Tn*Bgl3B [28], are shown with a yellow background. The blue underlining below the *Tn*Bgl3B sequence indicates domains 1, 2, and 3 of *Tn*Bgl3B identified by X-ray crystallography [28].

Strain KB0549 grown on medium 1 (see “Bacterial strains” section of Materials and methods) intracellularly produced an enzyme (PSTG), which was capable of degrading STG to produce sesaminol (specific activity, 1.1 nkat/mg protein). HPLC analysis of the reaction mixture with crude extract of the KB0549 cells showed that hydrolysis of STG was accompanied by an increase in sesaminol, with transient formation of 6-SDG, SMG, and a very small amount of 2-SDG (Fig. 2), as confirmed by co-chromatography using authentic samples. When strain KB0549 cells were grown in a medium (pH 7.0) containing 1% soluble starch as the sole carbon source (medium 2, see “16S rRNA sequence analysis” section of Materials and methods), a crude extract of the cells displayed only a low level of PSTG activity (specific activity, 0.08 nkat/mg protein), suggesting that PSTG was an inducible enzyme. Preliminary studies showed that PSTG activity was co-eluted with pNP-β-Glc-hydrolyzing activity during several different chromatographies, and thus could conveniently be identified on the basis of its pNP-β-Glc-hydrolyzing activity during purification. PSTG (40 µg) was finally purified to homogeneity, as judged by silver staining, after five chromatographic steps as described in the Materials and methods section. The purified enzyme gave a single protein band with an approximate molecular mass of 80 kDa (Fig. 3A). The N-terminal amino acid sequence of the purified PSTG, determined by automated Edman degradation, was Ser-Glu-Arg-Arg-Asp-Leu-Lys-Ala-Ile-Ser-Gln-Met-Thr-Leu-Glu-Glu-Lys-Ala-Ser (termed sequence 1, Fig. 4). The internal amino acid sequences of the purified protein were also determined as described in Materials and methods, as follows: Val-Asn-Gly-Glu-Tyr-Ala-Ala-Glu-Asn-Glu-Arg-Leu (sequence 2), Pro-Thr-Arg-Leu-Asp-Asp-Ile-Val-Phe-Glu (sequence 3), Leu-Ala-Glu-Thr-Phe- Pro-Val-Gln-Leu-Ser-Asp-Asn (sequence 4), and Leu-Arg-Gly-Met-Ile-Pro-Phe- Gly-Glu-Thr (sequence 5).

### Gene Cloning and Phylogenetics of PSTG

The PCR primers were designed on the basis of the amino acid sequences determined for the purified PSTG, and PCR was executed using genomic DNA of strain KB0549 as a template. A DNA fragment of 1.1 kbp was amplified. Sequences of its unknown flanking regions were clarified using the genomic walking method with an annealing control primer method [20]. Finally, a 3.1 kbp DNA fragment was obtained, which contained an open reading frame encoding a protein (Genbank/EMBL/DDBJ accession number, AB567660; UniProtKB/TrEMBL accession number, D6RVX0) of 753 amino acids with a predicted molecular mass of 83,298. The internal amino acid sequences determined for the enzyme purified from strain KB0549 (sequences 2, 3, 4, and 5) were identified at positions 204–215, 357–366, 497–508, and 701–710, respectively (Fig. 4).

**Figure 5:**
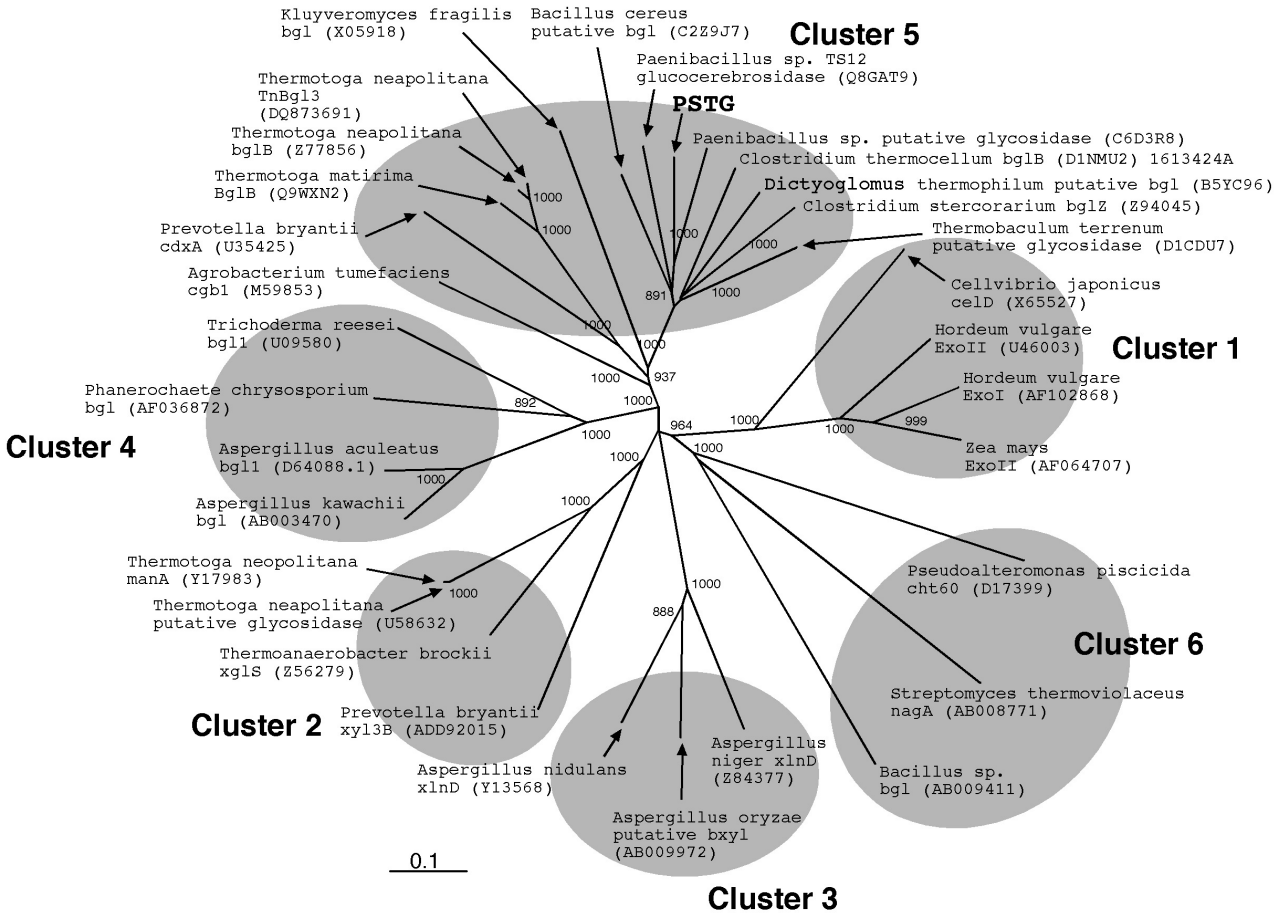
Non-rooted phylogenetic tree of GH3 family glycosidases. Enzyme names are shown with their DDBJ/EMBL/Genbank accession numbers (*parenthesized*). The tree was constructed from a CLUSTALW program multiple alignment [32] using a neighbor joining method [33]. Bar = 0.1 amino acid substitutions/site. Numbers indicate bootstrap values greater than 800. Known clusters (clusters 1–6) of GH3 family [23] are shown with gray circles.

The deduced amino acid sequence of PSTG was similar to those of glycosidases belonging to the glycoside hydrolase (GH) family 3 [21], [22] and assigned to a member of the cluster 5 of the GH3 family [23] (Fig. 5). Among GH3 enzymes that have been biochemically characterized so far, the highest sequence similarity was found with a glucocerebrosidase of *Paenibacillus* sp. TS12 (63% identity, see also Fig. 5) [24], [25]. Sequence similarities to other GH3 β-glucosidases were as follows: β-glucosidase of *Clostridium stercorarium* (O08331, 61%) [26], β-glucosidase of *Kluyveromyces marxianus* (X05918, 40%) [27], and *Tn*Bgl3B β-glucosidase of *Thermotoga neapolitana* (ABI29899, 37%) [28] (Fig. 5). The N-terminal sequence determined for the enzyme purified from strain KB0549 was located at positions 2–20 of the deduced amino acid sequence, which appeared to contain no signal sequence for secretion, as predicted by a computer program (PSORTb v.3.0; http://www.psort.org/psortb/) [29], and this was consistent with the fact that PSTG was an intracellular enzyme (see above).

### Molecular Properties of the Recombinant Enzyme

The *PSTG* gene was expressed as a catalytically active protein in *E. coli* cells. The recombinant enzyme (termed rPSTG) was purified to homogeneity by Ni^2+^-affinity chromatography (Fig. 3B). The native molecular mass of the purified rPSTG was estimated to be 310 kDa by gel filtration chromatography on the Superdex 200. Judging from the calculated molecular mass of protein subunits (see above), the recombinant enzyme likely exists as a tetrameric protein.

**Figure 6:**
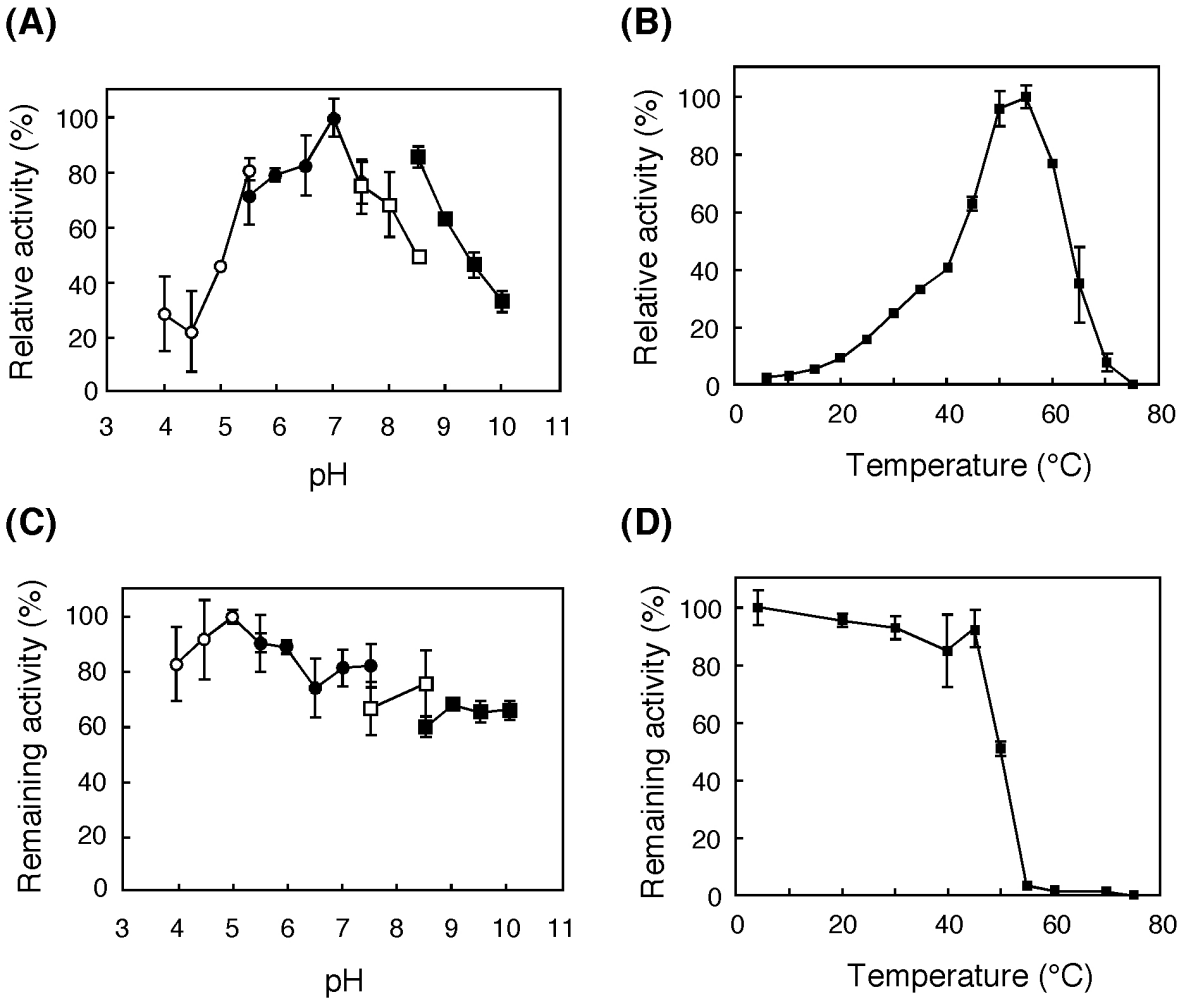
Effects of pH and temperature on enzyme activity (A and B) and on the stability (C and D) of rPSTG. The buffers used were sodium acetate (*open circles*), potassium phosphate (*closed circles*), HEPES-NaOH (*open rectangles*), and glycine-NaOH (*closed triangles*). The results are presented as the average of three determinations ±SD.

The rPSTG was active over a pH range of 5.5–9.0, with maximum activity at 7.0 (at 37°C) (Fig. 6A). This optimum pH for activity was similar to those of other GH3 β-glucosidases of the same bacterial genus (*i.e.,* TS12 glucocerebrosidase) [24], [25] and was higher than those of GH3 β-glucosidases of fungal and other bacterial origins. For example, β-glucosidases (BG_S_ and BG_3_) of *Aspergillus niger* show an optimum pH for activity between pH 4.0–4.5 [30], and *Tn*Bgl3B (see above) displays the highest activity at pH 5.6 [28]. The enzyme displayed the highest activity at 55°C under the conditions of assay method II (Fig. 6B). rPSTG was stable at pH 4–10 (at 4°C for 15 h, Fig. 6C) and below 45°C (at pH 6.5 for 30 min) (Fig. 6D). Thermostability of rPSTG was significantly lower than that of *Tn*Bgl3B, which is stable even at 90°C [28]. This is consistent with the fact that strain KB0549 was a mosophile that optimally grows at 37°C while *Thermotoga neapolitana*, the *Tn*Bgl3B producer, is a hyperthermophile that optimally grows at 77°C or higher temperatures [31].

### Enzymatic Hydrolysis of STG

**Figure 7:**
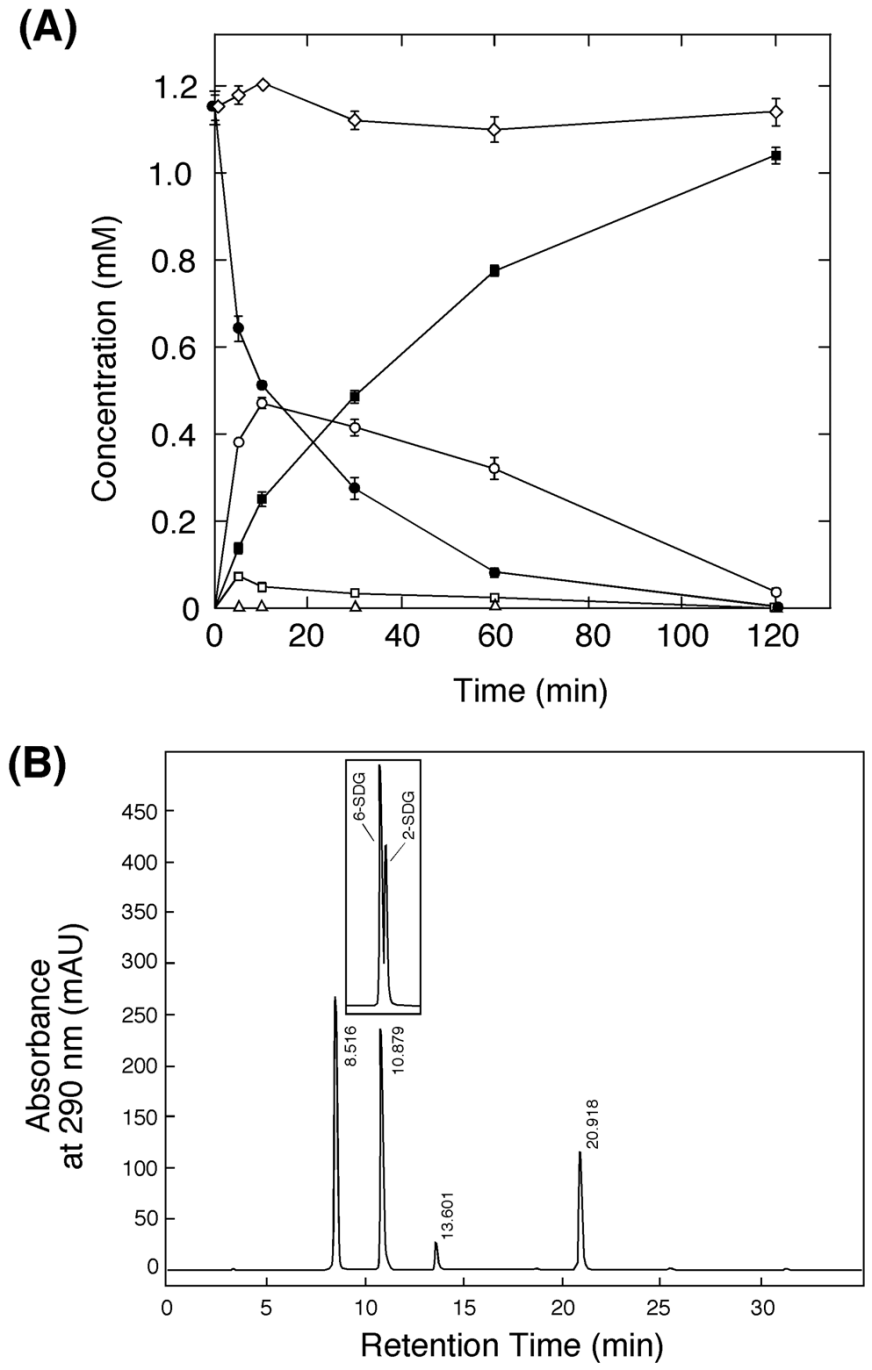
Analysis of the course of sesaminol production from STG catalyzed by rPSTG. (**A**) rPSTG (0.15 µM monomer protein) was reacted with 1.15 mM STG (initial concentration, 1.15 mM) at pH 7.0 and 37°C. During the reaction, concentrations of STG (*closed circles*), 6-STG (*open circles*), 2-STG (*open triangles*), SMG (*open squares*), and sesaminol (*closed squares*) in the reaction mixture were found by HPLC. *Open diamonds* show concentrations of STG in the mixture without enzyme. The results are presented as the average of three determinations ±SD. (**B**) Reversed-phase HPLC analysis of a reaction mixture. Retention times are: STG, 8.52 min; 6-SDG, 10.88 min; SMG, 13.6 min; and sesaminol, 20.92 min. *Inset* shows co-chromatography of 6-SDG and 2-SDG (11.08 min).

The course of the hydrolysis of STG (initial concentration, 1.15 mM) catalyzed by rPSTG (0.15 µM monomer protein) at pH 7.0 and 37°C was monitored by analytical reversed-phase HPLC. Hydrolysis of STG was accompanied by an increase in sesaminol and glucose, with transient formation of 6-SDG (Fig. 7A), as confirmed by co-chromatography with authentic samples of 6-SDG and 2-SDG (Fig. 7B, *inset*). A small amount of SMG was also identified during the reaction (Fig. 7B). The course of STG hydrolysis catalyzed by the rPSTG was very similar to those observed with the crude extract of the KB0549 cells (Fig. 1), transiently producing 6-SDG as a major intermediate during the reaction, and this strongly suggested that the cloned enzyme was responsible for the STG-hydrolyzing activity of the KB0549 cells. Mass spectroscopic analysis showed the absence of dimers and trimers of glucose in the reaction mixture. Stereoisomers of sesaminol (2-episesaminol, 6-episesaminol, and diasesaminol) were not identified in the reaction mixture. After 2 h, STG could be almost quantitatively converted to sesaminol under the conditions used (Fig. 7A). Stoichiometric studies using this mixture also showed that production of 1.0 mole of sesaminol from STG was accompanied by the formation of 3.3 mole of glucose. *k*_cat_ and *K*_m_ values of rPSTG for hydrolysis of STG were determined to be 9.3±0.8 s^−1^ and 1.4±0.4 mM, respectively, by means of initial velocity analysis at pH 7.0 and 37°C. Relative activity of hydrolysis of 2-SDG and 6-SDG (initial concentration, 1.15 mM) at pH 7.0 and 37°C was 124% and 53%, respectively, with the activity of STG hydrolysis taken to be 100%.

### Specificity Studies

To examine sugar substrate specificity of rPSTG, the ability of the enzyme to hydrolyze a variety of pNP-glycosides was examined at pH 7.0 and 37°C (Table 1). pNP-β-Glc was the best of the substrates tested, with *k*_cat_ and *k*_cat_/*K*_m_ values of 44±0.2 s^−1^ and 426 s^−1^mM^−1^, respectively. These values were lower than the values reported for *Tn*Bgl3B (129±3 s^−1^ and 2002 s^−1^ mM^−1^, respectively, at pH 5.6, 90°C) [21]. Although the *k*_cat_ value for pNP-β-D-xylopyranoside (69 s^−1^) was larger than that of pNP-β-Glc, *k*_cat_/*K*_m_ for pNP-β-D-xylopyranoside was only 0.4% of the value of pNP-β-Glc due to a very large *K*_m_ value (36 mM). pNP-β-D-cellobioside also acted as a poor substrate (relative *k*_cat_/*K*_m_, 0.5% of the value for pNP-β-Glc), although its *K*_m_ value was comparable with the value for pNP-β-Glc. Stoichiometric studies showed that the production of 1.0 mol of *p*-nitrophenol from pNP-β-cellobioside was accompanied by the formation of 1.7 mol of glucose. Moreover, mass spectrometric analysis of the reaction mixture showed the absence of glucose dimers, suggesting that PSTG was able to cleave the β-1,4-glucosidic linkage, albeit slowly (see also below). pNP-β-D-Galactopyranoside, pNP-β-D-fucopyranoside, and pNP-*N*-acetyl-β-D-glucosaminide were very poor substrates, and pNP-α-glucopyranoside was inert as a substrate of rPSTG. These results suggest that rPSTG specifically acts on the β-glucosidic linkage.

**Table 1:** Substrate specificity of rPSTG.

Substrate^a^	*k*_cat_ (s^−1^)	*K*_m_ (mM)	*k*_cat_/*K*_m_ (s^−1^/mM)
pNP-β-D-Glc	44.3±0.21	0.10±0.01	426
pNP-β-D-cellobiose	0.47±0.03	0.23±0.04	2.04
pNP-β-D-xylopyranoside	69	35.7±12.2	1.93
pNP-β-D-fucopyranoside^b^			0.23
pNP-β-D-galactopyranoside	2.06±0.07	29.0±7.0	0.071
pNP-N-acetyl-β-D-glucosaminide^b^			4.55×10^−4^
pNP-α-glucopyranoside	0	nd	nd

a pNP, *p*-nitrophenyl.

b A linear relationship between initial velocity (*v*) and substrate concentration [S] was obtained in the range of [S] examined (up to 10 mM), suggesting that the *K*_m_ value for the substrate should be significantly higher than 10 mM, because the enzyme-catalyzed reactions proceeds with first-order kinetics under the conditions of [S]<<*K*_m_ as described by *v* = (*k*_cat_/*K*_m_)[S], where *k*_cat_/*K*_m_ is the first-order rate constant. Thus, the ratio *k*_cat_/*K*_m_ could be determined from slope of *v* versus [S] plots.

nd, not determined.

We further examined the ability of rPSTG to hydrolyze sophorose, cellobiose, and gentiobiose (final concentration, 10 mM), which are dimers of glucose containing β-1,2-, β-1,4- and β-1,6-glucosidic linkages, respectively. The results showed that the enzyme displayed the highest activity toward sophorose (14±4 nkatal/mg), followed by cellobiose (2.6±1.6 nkatal/mg) and gentiobiose (1.2±0.6 nkatal/mg). It must be noted that, despite the relatively high sequence similarity of this enzyme to TS12 glucocerebrosidase of *Paenibacillus* [28], rPSTG displayed no detectable glucocerebrosidase activity, as assayed using D-glucosyl-β-1,1′-*N*-octanoyl- Δ-*erythro*-sphingosine.

## Discussion

Specificity studies clearly showed that PSTG was highly specific for the β-glucosidic linkage and thus considered to be a β-glucosidase. Consistently, PSTG shares primary structural characteristics that are important for the specificity and catalytic mechanism of GH3 β-glucosidases. For example, glucose-binding amino acid residues involved in subsite –1 in the crystal structure of the *Tn*Bgl3B β-glucosidase, as well as those of the TS12 glucocerebrosidase of *Paenibacillus* sp., were strictly conserved in the primary structure of PSTG (Asp46, Leu118, His155, Arg165, Met198, Tyr201, Trp234, and Ser352; Fig. 4). Catalytic amino acid residues that were identified in the TS12 glucocerebrosidase [24] and crystal structure of the *Tn*Bgl3B β-glucosidase [28] were also conserved (Asp233 and Glu421; see Fig. 4). However, PSTG was distinguished from other β-glucosidases in terms of its specificity for glucosidic linkage types–it displays high activity toward β-1,2-glucosidic linkage, which greatly exceeds the activities toward β-1,4- and β-1,6-glucosidic linkages. Thus, rPSTG can be denoted “β-1,2-glucosidase”. This unique specificity appears to be related to the ability of the enzyme to efficiently decompose STG (see below). Previously, SMG, 2-SDG and STG were identified in sesame seeds, and SMG and 2-SDG were reported to be highly resistant to hydrolysis by β-glucosidase [6]. In this context, PSTG is unique because it is capable of hydrolyzing all three of the β-glucosidic linkages in the STG molecule, and it displayed a higher preference for β-1,2-glucosidic linkage than for β-1,6-glucosidic linkage, resulting in the transient formation of 6-SDG (not 2-SDG).

**Figure 8:**
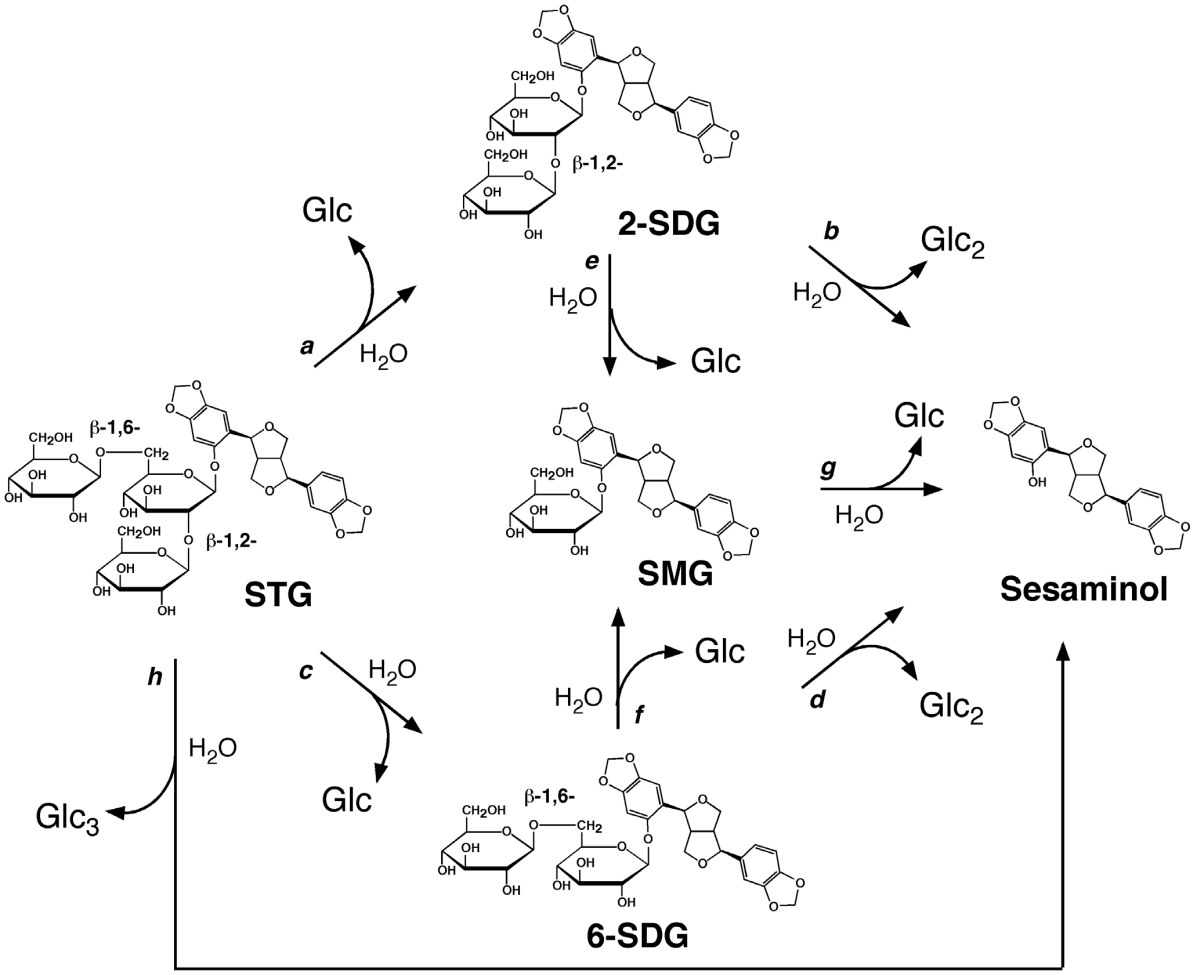
Possible pathways of enzymatic production of sesaminol from STG.

Figure 8 shows the possible reaction pathways for the enzymatic production of sesaminol from STG. These pathways include the one-by-one removal of glucose moieties (in either sequential or random manner, where SDG(s) and/or SMG will be transiently produced as the intermediates). These also include the one-step (*arrow h*) and two-step removal (*arrows a/b or arrows c/d*) of the sugar chain; however, these one- and two-step pathways were highly unlikely because no detectable amount of dimers and trimers of glucose was observed during the reaction, as analyzed by mass spectrometry. All of the results obtained in this study consistently suggest that the sesaminol production from STG catalyzed by rPSTG mainly proceeds with the one-by-one removal of glucose moieties, as shown by *arrows c/f/g*, where the hydrolysis of β-1,2-glucosidic linkage in the STG molecule takes place first to result in the transient accumulation of 6-SDG. However, the relative activity of 6-STG (53% of STG hydrolysis) was not significantly lower than that of 2-SDG (124%), suggesting that the rate of the enzymatic cleavage of the β-1,6-glucosidic linkage in the STG molecule (*arrow a*) should not be significantly slower than that of the β-1,2-glucosidic linkage (*arrow c*). Thus, the order of the removal of glucose moieties in the STG molecule might not be strictly compulsory–the enzymatic production of sesaminol from STG might also in part proceed with the minor pathway shown by *arrows a/e/g*, where only a negligible amount of 2-SDG accumulated in the reaction mixture.

Finally, the fact that 6-SDG accumulates during the PSTG-catalyzed production of sesaminol from STG suggests that the supplemental addition of a “β-1,6-glucosidase” to the reaction mixture will further enhance the efficiency of the production of sesaminol from STG. Such “β-1,6-glucosidase” activity is easily available from inexpensive commercial sources (*e.g*., cellulases). Examination of the large-scale enzymatic production of sesaminol from sesame oil cake is currently underway.
